# The association of genitourinary cancer among Saudi patients with gastrointestinal stromal tumors and a systematic literature review

**DOI:** 10.1038/s41598-023-28060-x

**Published:** 2023-03-15

**Authors:** Raouf Seyam, Kenan Abou Chaer, Ahmed Abouelkhair, Arwa Almouh, Othman Alzahrani, Ahmed Gamal Sayed, Mohammad Alghafees, Razan A. Alhamidi, Ali Al-Gonaim, Turki Al-Hussain, Tarek Mahmoud Amin, Waleed Altaweel

**Affiliations:** 1 Department of Urology, King Faisal Specialist Hospital and Research Center, Riyadh, Saudi Arabia; 2 College of Medicine, Alfaisal University, Riyadh, Saudi Arabia; 3 College of Medicine, King Saud Bin Abdul-Aziz University for Health Sciences, Riyadh, Saudi Arabia; 4 Department of Pathology and Laboratory Medicine, King Faisal Specialist Hospital and Research Center, Riyadh, Saudi Arabia; 5 Surgical Oncology Department, Oncology Center, King Faisal Specialist Hospital and Research Center, Riyadh, Saudi Arabia

**Keywords:** Urological cancer, Gastrointestinal cancer

## Abstract

The gastrointestinal tract's most commonly occurring primary mesenchymal tumor is the gastrointestinal stromal tumor (GIST). However, few cases worldwide were reported associated with renal cell carcinoma (RCC). Therefore, we aimed to identify the association of genitourinary tumors in patients with GIST in our tertiary care hospital in Saudi Arabia and compare it to the literature. We identified all patients in the pathology department database with the diagnosis of GIST. We excluded duplicate and recurrent cases. We examined patients’ files for the presence of RCC, adrenal tumors, or other genitourinary cancer. A systematic review of the association was conducted. From 2003 to 2020, 170 patients had a histopathologic diagnosis of primary GIST, 100 men and 70 women, median age of 57 (range 9–91) years at the time of diagnosis. The site of primary GIST was gastric 103, small bowel 43, mesenteric 5, omentum/peritoneum 7, abdomen 4, isolated adrenal 1, and other 7. Six patients had associated primary genitourinary cancer. Three patients had RCC (two clear cell RCC and one radiologic diagnosis only), and three had adrenal tumors (one adrenal carcinoma, one an isolated adrenal GIST, and one pheochromocytoma). In addition, two patients had a tumor invading the urinary bladder. Although the cohort included 63 men aged 60 or above (median 71 ± 8.7 years, range 60–94), none demonstrated clinical prostatic carcinoma. Data was compared to 69 systematic review articles. We report the rare association between GIST tumors and primary genitourinary cancer, mainly RCC and adrenal tumors. Also, we identified a secondary invasion of the urinary bladder. Unlike the reported series, none of the older male patients had clinical prostate cancer.

## Introduction

The most commonly occurring primary mesenchymal tumor in the gastrointestinal tract is a gastrointestinal stromal tumor (GIST). Since the tumor has been molecularly characterized only recently, it is not easy to determine its actual frequency; however, population-based studies suggest that the annual incidence per million population is between 11 and 15^1^. Most GISTs seemingly occur sporadically, but nearly 5% of all GISTs are linked with inheritable mutations or syndromes.

Several studies reported the association between GIST and second primary malignancies. Next to other gastrointestinal tumors, genitourinary (GU) cancer is associated with GIST^2–6^. In addition, the association is often related to KIT mutations^7,8^.

The most reported associated primary GU tumors are prostatic adenocarcinoma, renal cell carcinoma (RCC), urothelial cancer, seminoma, and adrenal tumors. Therefore, we set out to identify the prevalence of genitourinary tumors in our hospital patients with histopathological diagnosis of GIST.

## Material and methods

This retrospective study included all patients diagnosed with GIST in the pathology department database. The association with RCC and other genitourinary tumors was identified. We defined genitourinary cancer as any cancer involving the kidney, adrenals, urinary bladder, prostate, testis, or penis. We cross-examined the RCC database for the association of GIST. Inclusion criteria included any patient with a histopathologic diagnosis of primary GIST. We excluded duplicate pathology reports of the same patient and cases with GIST recurrences. We excluded tumors of the ovaries and the female genital tract. Descriptive statistics were used. A systematic review literature search was made in Medline/PubMed. The search syntax was generated to combine MeSH terms of “GIST” OR “Gastrointestinal Stromal Tumors” AND MeSH terms of “Neoplasms, Second Primary” OR “Neoplasms, Multiple Primary” OR a text word indicating genitourinary cancer (renal, adrenal, urothelial, bladder, prostatic, OR testicular cancer) OR a genitourinary organ (kidney, adrenal, prostate, urinary bladder, ureter, OR testis). A filter was added restricting the search to the English language and humans.

### Ethical approval

This retrospective chart review study involving human participants was following the ethical standards of the institutional and national research committee and with the 1964 Helsinki Declaration and its later amendments. The Human Investigation Committee (IRB) and Research Ethics Committee of King Faisal Specialist Hospital and Research Center approved this study.

### Consent for publication

The Human Investigation Committee (IRB) and Research Ethics Committee of King Faisal Specialist Hospital and Research Center waived the consent because of the study's retrospective nature, and all the procedures being performed were part of the routine care.

## Results

Our patients: From 2003 to 2020, 170 patients had a histopathologic diagnosis of primary GIST, 100 men and 70 women, median age of 57 (range 9–91) years at the time of diagnosis. There were 144 patients with GIST-only tumors (84.7%), 26 with other primary cancer, including six patients with primary genitourinary cancer and two with GIST direct invasion of the urinary bladder (Table 1). Primary GIST was most commonly gastric, spindle cell type, had a low mitotic rate and CD117, DOG1, or both immunostainings. Few cases underwent genetic mutation analysis, and none had genetic counseling. Only one-third of patients were treated with imatinib. Patients with another primary cancer had significantly lower TNM stage and less epithelioid histopathology than GIST-only patients. No significant differences were observed amongst patients with GU vs. no GU cancer.

**Table 1:** Demographic and pathological characteristics of patients with GIST and associated cancer.

Continuous variables	All		Only GIST		Other 1ry cancer			No GU cancer		1ry GU cancer		
	n	Med (range)	n	Med (range)	n	Med (range)		n	Med (range)	n	Med (range)	
Age at diagnosis years	170	57 (9–91)	144	58 (9–91)	26	56 (16–81)		164	58 (9–91)	6	51 (36–55)	
GIST Size cm	161	6 (0.3–36)	135	7 (0.5–36)	26	2 (0.3–27)		155	6.5 (0.3–36)	6	2.6 (0.5–8)	

Categorical variables		n (%)		n (%)		n (%)	P*		n (%)		n (%)	P**
Sex												
Male		100 (58.8)		86 (50.6)		14 (8.2)	0.666		95 (55.9)		5 (2.9)	0.211
Female		70 (41.2)		58 (34.1)		12 (7.1)			69 (40.6)		1 (0.6)	
Total		170 (100)		144 (84.7)		26 (15.3)			164 (96.5)		6 (3.5)	
GIST primary site												
Gastric		102 (60)		86 (50.6)		16 (9.4)	0.382		100 (58.8)		2 (1.2)	0.062
Small intestine		47 (27.6)		41 (24.1)		6 (3.5)			45 (26.5)		2 (1.2)	
Omentum/peritoneum/mesenteric		12 (7.1)		11 (6.5)		1 (0.6)			12 (7.1)		0 (0.0)	
Other		9 (5.3)		6 (3.5)		3 (1.8)			7 (4.1)		2 (1.2)	
GIST stage												
T1		25 (14.7)		11 (6.5)		14 (8.2)	0.000		22 (12.9)		3 (1.8)	0.105
T2		47 (27.6)		42 (24.7)		5 (2.9)			45 (26.5)		2 (1.2)	
T3		44 (25.9)		42 (24.7)		2 (1.2)			43 (25.3)		1 (0.6)	
T4		45 (26.5)		40 (23.5)		5 (2.9)			45 (26.5)		0 (0.0)	
N0		34 (20.0)		29 (17.1)		5 (2.9)	0.286		32 (18.8)		2 (1.2)	0.670
N1		5 (2.9)		3 (1.8)		2 (1.2)			5 (2.9)		0 (0.0)	
M0		13 (7.6)		11 (6.5)		2 (1.2)	0.000		13 (7.6)		0 (0.0)	1.000
M1		25 (14.7)		21 (12.4)		4 (2.4)			24 (14.1)		1 (0.6)	
Histopathology												
Spindle cell		85 (50.0)		70 (41.2)		15 (8.8)	0.028		80 (47.1)		5 (2.9)	0.718
Epithelioid type		16 (9.4)		13 (7.6)		3 (1.8)			16 (9.4)		0 (0.0)	
Mixed epithelioid and spindle		10 (5.9)		10 (5.9)		0 (0.0)			10 (5.9)		0 (0.0)	
Other		2 (1.2)		0 (0.0)		2 (1.2)			2 (1.2)		0 (0.0)	
Mitotic rate												
≤ 5/50 HPFs		97 (57.1)		83 (48.8)		14 (8.2)	0.647		94 (55.3)		3 (1.8)	0.729
> 5/50 HPFs		39 (22.9)		34 (20.0)		5 (2.9)			38 (22.4)		1 (0.6)	
Immune staining												
CD117 (c-kit) positive		97 (57.1)		85 (50.0)		12 (7.1)	0.070		94 (55.3)		3 (1.8)	0.391
CD117 (c-kit) and DOG1 positive		33 (19.4)		23 (13.5)		10 (5.9)			30 (17.6)		3 (1.8)	
DOG-1 positive		12 (7.1)		11 (6.5)		1 (0.6)			12 (7.1)		0 (0.0)	
Treated with Imatinib												
Yes		56 (32.9)		51 (30.0)		5 (2.9)	0.079		54 (31.8)		2 (1.2)	0.645
No		114 (67.1)		93 (54.7)		21 (12.4)			110 (64.7)		4 (2.4)	

*GIST* gastrointestinal tumor; *GU* genitourinary; *HPF* high power field; *Med* median;* P* Fisher's Exact Test 2-tail; P* comparison only GIST vs other primary cancer; P** comparison GU cancer vs no GU cancer.

**Figure 1:**
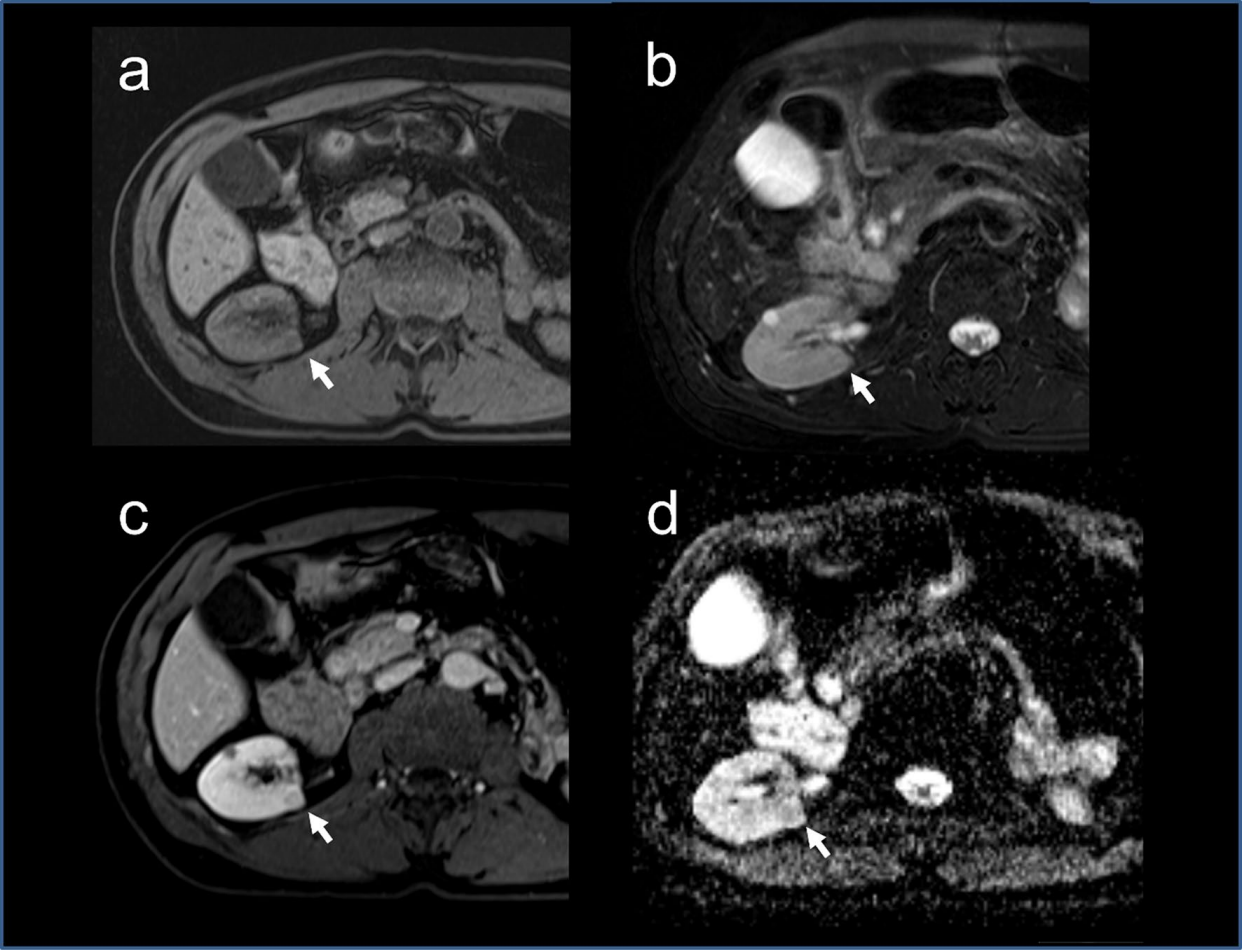
Radiological diagnosis of RCC in a GIST patient. MRI showing a coronal section of the abdomen with renal lesion (arrows). (**a**) T1 FS image showing a mild low signal intensity of a right posterior 8 mm renal mass. (**b**) T2 FS image showing increased intensity of the renal lesion. (**c**) T1 FS image showing hyperenhancement of the lesion after contrast injection. d. ADC MAP showing diffuse restriction of the lesion.

**Figure 2:**
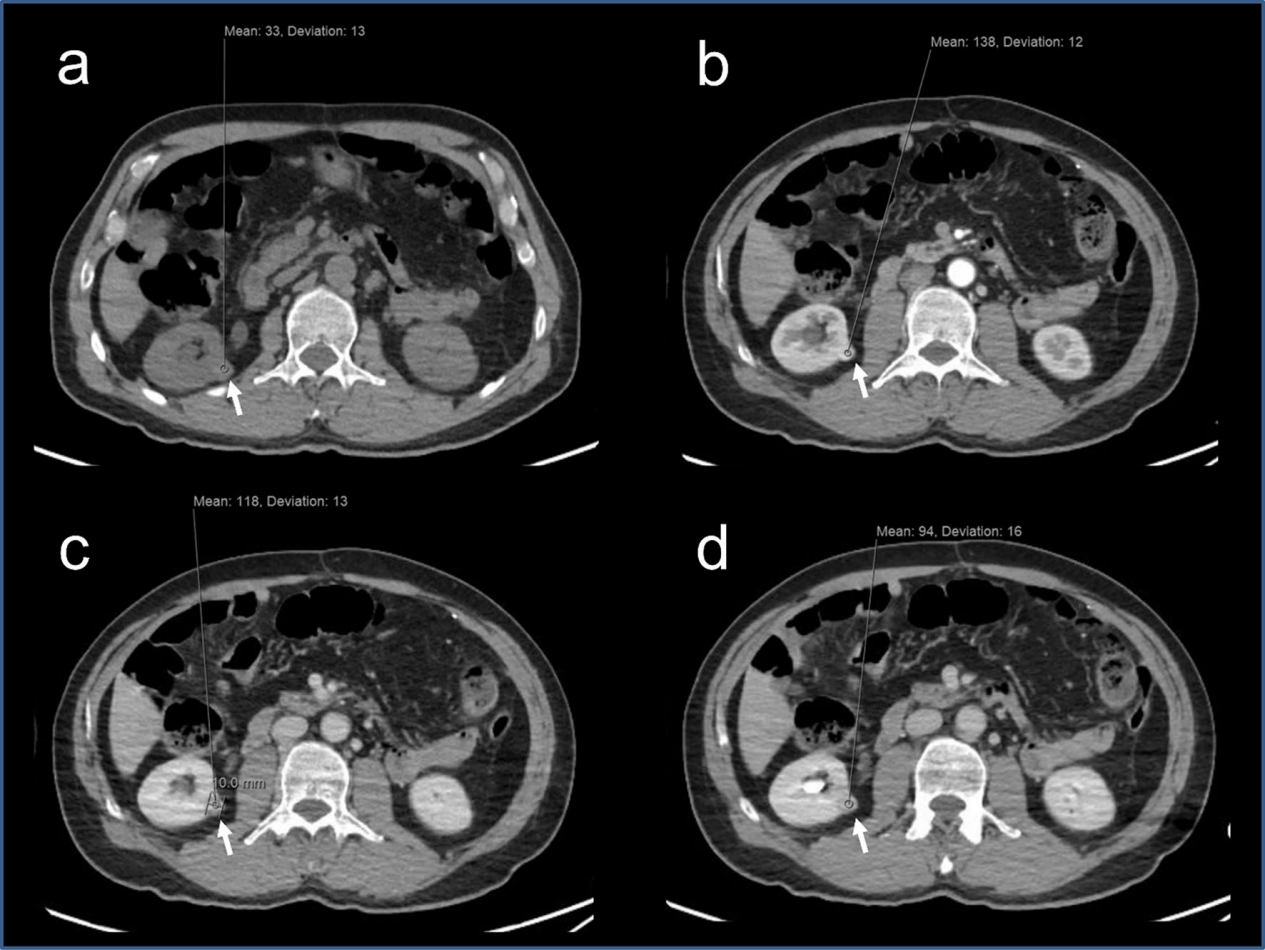
CT scan of the abdomen at follow up of the same patient after 2 years showing Hounsfield density measurement of the renal tumor consistent with right RCC (arrows). (**a**) CT without contrast, lesion density 33 ± 13. (**b**) Arterial phase, the density increased to 138 ± 12. (**c**) Venous phase the density decreased to 118 ± 13. (**d**) Delayed phase the density decreased to 94 ± 16.

All GIST – GU tumors in our cohort were synchronously discovered during the investigation and treatment of the GU cancer. Two patients had associated clear cell RCC. One tumor was a renal mass with radiological characteristics suggesting papillary RCC (Fig. 1). The tumor was 8 mm in diameter. As there was no indication for surgery, it was followed up by observation with no histological diagnosis. Follow-up CT after two years was consistent with RCC (Fig. 2). Cross-examination of the RCC database retrieved the two histologically proven RCCs. Three cases had adrenal tumors (one adrenal carcinoma, one an isolated adrenal GIST, and one pheochromocytoma). One tumor presented as a urinary bladder mass and was evaluated by transurethral resection. The bladder tumor on histopathology was GIST due to direct invasion from the colon that was not clear on the preoperative radiological diagnosis. Another patient had a urinary bladder invasion by GIST arising from the mesentery.

Of note is that the GIST cohort included 63 male patients who were 60 years or older (median 71 ± 8.7 years, range 60–94). None of them, however, had prostate cancer.

Systematic review: A total of 429 articles were identified, and abstracts were reviewed. Further selection was based on information indicating the presence of GIST in association with genitourinary cancer or organ. Duplicate publications were removed. We found 59 articles meeting the selection criteria and added ten more from references in these articles. A total of 69 articles were reviewed. Seventeen articles reported the prevalence of genitourinary cancer associated with GIST ranging 2.1–15.6% (Table 2)^7–23^. Prostatic adenocarcinoma was the most common primary genitourinary cancer (2.1–14%), followed by RCC (0.6–11.4%). Rarely seminoma^8,11,16,21^ and a single case of adrenal neuroblastoma were reported^19^. Additional 52 articles included case reports of the association (Table 3)^2,3,5,24–55^, or a primary genitourinary organ GIST (Table 4)^56–72^.

**Table 2:** Reported series of GU cancer associated with GIST.

Reference	GIST (n)	1ry GU cancer (n)	%	GU tumor, n (%)
Current study	170	6	3.5	RCC 3 (1.8), adrenal 3 (1.8)
Szczepaniak and Nasierowska-Guttmejer^9^	76	4	5.3	CCRCC 1 (1.3), prostate adenocarcinoma 3 (3.9). Age 74–77 yrs
Shen et al.^10^	8511	310	3.6	Prostate cancer 236 (2.77) urinary system cancer 74 [0.87]
Comandini et al.^11^	145	6	4.1	Seminoma 1 (0.69), Prostate cancer 3 (2.1), Renal cancer 2 (1.4)
Petrelli et al.^12^	19,627	3%		Prostate cancer (14%), Kidney cancer (4.35%)
Fernández et al.^13^	104	8	7.7	CCRCC 1 (1), CrRCC 1 (1), bladder urothelial cancer 2 (1.9), prostate adenocarcinoma 3 (2.9), age 68–74 yrs, angiomyoliposarcoma 1 (1)
Mayr et al.^8^	188	17	9.0	Renal 8 (11.4), prostate 6 (8.6), urothelial 2 (2.9), seminoma 1 (1.4)
Mendonca et al.^14^	405	9	2.2	Kidney cancer 9 (2.2%); (4/9, papillary RCC; 4/9 CRCC, 1/9 CrRCC)
Aghdassi et al.^15^	104	8	7.7	Prostate cancer 6 (5.8), RCC 1 (1), urothelial cancer 1 (1). Mean age 66.9 yrs
Hechtman et al.^7^	260	15	5.8	Prostate 11 (4.2), renal 4 (1.5). Median age 65–66 yrs
Kramer et al.^16^	836	59	7.1	Prostate cancer 38 (4.5), seminoma 1 (0.1), RCC 12 (1.4), urothelial cancer 9 (1.1). Median age 67.9 yrs
Murphy et al.^17^	6112	249	4.1	Bladder adenocarcinoma 3 (0.05), prostate adenocarcinoma 184 (3), RCC 35 (0.6), bladder TCC 27 (0.4). Age = > 60, 61/5
Vassos et al.^18^	86	5	5.8	Prostatic adenocarcinoma 3 (3.5), urothelial carcinoma 1 (1.2), RCC 1 (1.2). Mean age 70 yrs
Gonçalves et al.^19^	101	3	3.0	CCRCC 1 (1), prostate adenocarcinoma 1 (1), adrenal neuroblastoma 1 (1)
Pandurengan et al.^20^	783	62	7.9	Cancer of prostate 28 (3.6), kidney 12 (1.5), bladder 6 (0.8), testis 1 (0.9), ureter 1 (0.9)
Agaimy et al.^21^	486	76	15.6	Carcinomas of prostate 43 (9), kidney 27 (6), seminoma 6 (1)
Agaimy and Wuensch^22^	97	2	2.1	RCC 1, prostate cancer 1
Au et al.^23^	74	4	5.4	Papillary RCC (3), prostate carcinoma (1)

*CCRCC* clear cell renal cell carcinoma; *CrRCC* chromophobe renal cell carcinoma; *GIST* gastrointestinal tumor; *GU* genitourinary; *RCC* renal cell carcinoma; *TCC* transitional cell carcinoma.

**Table 3:** Case reports of GU cancer associated with GIST.

Reference	Setting	1ry GIST	1ry GU cancer (n)	Pathology
Arif et al.^24^	NF1	Jejunum	Adrenal	Pheochromocytoma
Vongsumran et al.^25^	NF1	Small intestine	Adrenal	Pheochromocytoma
Gorgel et al.^26^	NF1 (3 patients)	Small intestine	Adrenal	Pheochromocytoma
Vlenterie et al.^27^	NF1 (2 patients)	Gastric, jejunum	Adrenal	Pheochromocytoma
Carşote et al.^28^	NF1	Duodenum	Adrenal	Pheochromocytoma
Boguszewski et al.^29^	Acromegaly	Gastric	Adrenal	Pheochromocytoma
Hataya et al.^30^	NF1	Intestine	Adrenal	Pheochromocytoma
Kramer et al.^31^	NF1	Ileum	Adrenal	Pheochromocytoma
Teramoto et al.^32^	NF1 (2 patients)	Small intestine, mesentery	Adrenal	Pheochromocytoma
Nemoto et al.^33^	NF1	Small intestine	Adrenal	Pheochromocytoma
Bümming et al.^34^	NF1, Carney triad (2 patients)	GIST site NA	Adrenal	Pheochromocytoma
Kovecsi et al.^35^	Upper GI symptoms	Gastric	Adrenal	Carcinoma
Tansir et al.^36^	Carney's triad	Gastric	Adrenal	Adenoma
Huang et al.^37^	LUTS	Rectum	Prostate	Sarcoma
Wei et al.^38^	Prostate cancer	Jejunum	Prostate	Cancer
Watanabe et al.^39^	Hematuria	Rectum	Prostate	Adenocarcinoma
Przybylik-Mazurek et al.^40^	NF1	Duodenum	Prostate	Adenocarcinoma
Laurens et al.^41^	Prostate cancer	Gastric	Prostate	Adenocarcinoma
Rebegea et al.^42^	Prostate cancer	Gastric	Prostate	Adenocarcinoma
Healy et al.^43^	Prostate cancer	Rectum	Prostate	Adenocarcinoma
Waisbren et al.^44^	Breast cancer	Gastric	Prostate	Adenocarcinoma
Macías-García et al.^45^	Rectal bleeding, LUTS	Rectum	Prostate	Adenocarcinoma
Engin and Ustündağ^46^	Gastric tumor	Gastric	Prostate	Adenocarcinoma
Yaman et al.^47^	LUTS	Rectum	Prostate	Adenocarcinoma
Kalender et al.^48^	NA	GIST site NA	Prostate	Adenocarcinoma
Miettinen et al.^49^	SDH cancers	Gastric	Renal	Unclassified RCC
Gill et al.^3^	SDH mutation	Gastric	Renal	Unclassified RCC
Omeroglu et al.^50^	Acute abdomen	Gastric	Renal	RCC
Juric and Basic-Jukic^51^	Renal transplantation	GIST site NA	Renal	RCC
Reşorlu et al.^52^	Upper GI symptoms	Gastric	Renal	Papillary RCC
Lim and Wojcik^53^	Referral	Colon	Renal	Papillary RCC
Jiang et al.^2^	GI bleeding	Gastric	Renal	Chromophobe RCC
Jawiarczyk-Przybyłowska et al.^54^	Acromegaly	Gastric	Renal	CCRCC
Tao et al.^55^	Hematuria	Gastric	Renal	CCRCC
Dasanu et al.^5^	GI bleeding	Small intestine	Renal (bilateral)	Papillary RCC

*CCRCC* clear cell renal cell carcinoma; *GI* gastro-intestinal; *GIST* gastrointestinal tumor; *LUTS* Lower urinary tract symptoms; *NA* not available; *NF1* Neurofibromatosis type 1; *RCC* renal cell carcinoma; *SDH* succinate dehydrogenase.

**Table 4:** Case reports of primary GU organ EGIST.

Reference	Setting	1ry GIST
Abou Al-Shaar et al.^56^	Flank pain	Adrenal
Sereg et al.^57^	Abdominal pain	Adrenal
Li et al.^58^	LUTS	Prostate
Shen et al.^59^	LUTS	Prostate
Alabed^60^	Screening	Prostate
You and Zhang^61^	Pelvic mass	Prostate
Etit et al.^62^	Perineal pain	Prostate
Liu and Xu^63^	LUTS	Prostate
Huh et al.^64^	LUTS	Prostate
Liu et al.^65^	LUTS	Prostate
Zhou and Teng^66^	LUTS	Prostate
Zhang et al.^67^	LUTS, hematuria	Prostate
Ou et al.^68^	LUTS	Prostate
Loeb et al.^69^	LUTS	Prostate
Yinghao et al.^70^	Perineal pain	Prostate
Lee et al.^71^	LUTS	Prostate
Van der Aa et al.^72^	Retention	Prostate

*EGIST* extra gastrointestinal stromal tumor; *LUTS* lower urinary tract symptoms.

There were 35 case reports of an association between GIST and genitourinary cancer. The most common associations were prostatic cancer (12), adrenal pheochromocytoma (11), and RCC (10). Case reports of primary extra gastrointestinal stromal tumor (EGIST) affecting the genitourinary tract were 17. The most common genitourinary EGIST was affecting the prostate (n = 15). The radiological and pathological evidence suggested that the GIST was not an extension from the adjacent bowel. Two cases were reported from the adrenal; one was from our institution^56,57^.

## Discussion

The number of our patients ranks 9th among the 17 studies reporting the prevalence of GU cancer in GIST patients (Table 2). In contrast, we report a 3.5% association ranking our study among the four least reported associations. The lower rate in our patients is probably related to the absence of prostate cancer compared to others. Series that reported prostate adenocarcinoma had a mean and median age between 65 and 77^7,9,13,15,16^. In our study, 61.3% of men were 60 years or older (Table 5). Those men had a median age of 71 and ranged from 60 to 94. At this age range, we expected to identify some cases of prostate cancer, but we did not find any^73^. The discrepancy is probably related to the high incidence and prevalence of prostate cancer in USA cancer registries compared to the Saudi and worldwide population (Table 6)^74^. There is a marked discrepancy in the cancer prevalence of the genitourinary tract^74^. The prevalence rate per 100,000 population of cancers in the USA is threefold higher than the reported worldwide rate and seven times the Saudi Arabian rate (Table 6). Several factors, like geographical differences in genetic makeup, risk factors, early detection, reporting period, and reporting system, may explain these differences. Of note is that the reported prevalence of prostatic cancer is much higher in patient populations than in the community. For example, in the USA, the prevalence of prostate cancer in the community for men 60 years and above is 1.96%, whereas the corresponding prevalence in the Surveillance, Epidemiology and End Results (SEER) database is 4.8–16.7%^73,74^. As our report is in a patient cohort rather than a community-based study, we believe that the lack of prostate cancer detection is an actual difference compared to other western countries. These findings may indicate that the association between prostate cancer and GIST is not due to a common risk factor but rather a geographical difference for prostate cancer. Another discrepancy is the absence of primary urothelial cancer in our cohort. Others reported 0.9–2.9% prevalence associated with urothelial cancer^8,13,15–17,20^. This feature is also surprising as urothelial cancer is highly prevalent in Saudi Arabia, comparable to renal cancer (Table 6).

**Table 5:** Men 60-year-old and above in the current study and corresponding USA prostate cancer prevalence.

Age	Number of patients current study 2003–2020	Prevalence (SEER) 1975–2015^73^	Expected number of patients current study
60–69	29	4.8%	1.4
70–79	20	12.4%	2.5
80 +	14	16.7%	2.3
Total	63		6.2

**Table 6:** Estimated number of 5-year prevalent cases in 2020 of genitourinary tumors according to the geographical region, both sexes, all ages^74^.

Area	Saudi Arabia		USA		Worldwide	
Cancer	n	Proportions*	n	Proportions	n	Proportions
All cancers	81,548	234.2	5,296,046	1600	44,091,402	565.7
Prostate**	2696	13.4	812,431	496	4,956,901	126.1
Bladder	2708	7.8	269,259	81.3	1,720,625	22.1
Kidney	2733	7.9	213,695	64.6	1,207,547	15.5
Testis	855	4.2	41,706	25.5	296,686	7.5
Penis	12	0.06	5281	3.2	102,157	2.6

*Prevalence per 100,000 population.

**Prevalence in males only.

On the other hand, the prevalence of 1.8% of renal cancer is comparable to most studies (0.6–2.2%), except two reporting 4.35% and 11.4%^8,12^. Out of the three cases, two were CRCC, and the radiological features of the third were suggestive of papillary RCC. This is not surprising as papillary RCC prevalence in GIST patients is much proportionally higher^14,23^. The GISTs in our GU cancer patients were incidental during planned nephrectomy or adrenalectomy. The incidental GIST was small but otherwise pathologically comparable to GIST with no associated GU cancer (Table 1). No genetic analysis was available to link the two pathologies. Clinically, however, none of our patients had VHL or NF-1 disease.

Other unique features of this study involve a rarely reported association with adrenal tumors. Of interest is the primary GIST of the adrenal gland. There is only one reported adrenal EGIST other than the one reported from our institution (Table 4)^56,57^. There is no radiological evidence that the adrenal GIST was a direct or distant spread of another primary GIST lesion. We also report two adrenal tumors, one carcinoma, and the other pheochromocytoma, none reported in the reviewed prevalence series (Table 2). However, sporadic cases of 11 pheochromocytomas were reported and most associated with NF1, unlike our patient (Table 3). There is only one adrenal carcinoma case report in addition to the one in our series (Table 3)^35^. Rarely was urothelial cancer reported in association with GIST (Table 2). Although one case presented as a bladder tumor, unlike the other reports, the two bladder tumors we report were secondary to direct invasion of a primary intestinal GIST. In addition, unlike the reports, we did not find associated testicular tumors. The strong point of this study is that it includes a large cohort of patients with histopathology-proven GIST comparable to large international series. In addition, it highlights prevalence differences unique to our geographical location. The study's weakness is that it did not include molecular and genetic profiling of the tumors and its retrospective design.

## Conclusion

We report the rare association between GIST tumors and primary genitourinary cancer, mainly RCC and adrenal tumors. Also, we identified a secondary invasion of the urinary bladder. Unlike the reported series, none of the older male patients had clinical prostate cancer.

## Supplementary Information

Supplementary Information.

## Data Availability

Data generated or analyzed during this study are included in the supplementary file.
